# Midwives

**Published:** 1907-04

**Authors:** 


### MIDWIVES.

The word midwife is defined as a woman who delivers parturient women. The word obstetrician one who practices obstetrics. The word obstetrics as the art of managing childbirth cases; that branch of surgery which deals with the management of pregnancy and labor. From these three definitions it would seem that the midwife is one who delivers parturient women without consideration of the art of obstetrics; in other words, one who through familiarity with the lying-in-chamber and without any attempt at study or comprehension of the subject surgically, and from clinical experience alone, has gained certain proficiency with labor.

To any practitioner of medicine the mental attitude of one who through clinical experience alone has attained enough skill to impress her patient with the fact that she "knows a great deal" when the actual condition is one of colossal ignorance is apparent; and the idea of trusting the life of the mother and the unborn child to such a one is repugnant in the extreme. There can be no doubt that such a woman is capable of receiving the child at birth, tieing the cord and accepting the placenta, and everything is normal, no hemorrhage or sepsis, the patient will undoubtedly do well; but, in the case of complications either at birth or post-partem, and the discovery of latent kidney disease, that all these things should be neglected would seem to place the patient in terrible jeopardy. When one does a considerable amount of clinical gynecology and sees the conditions left years after delivery by the midwife, it certainly is a subject for careful consideration.

Where the obstetrical history of the patient from conception to the end of the parturient state is absolutely normal, and no infection is carried by the attendant, that patient is safe from trouble. Such a patient goes through a normal process and needs little or no care from any one; but, and this but is such a large one and affects so many hundreds and thousands of women, if there are any variations from the normal there is the need of skill and care second to none in any branch of medicine. It certainly would seem that if ever a woman needed good care it is during the time at which she will undergo the sorest trial of her life.

There is another side to the midwife, namely: the criminal side. It has become synonymous in one's mind that at the sound of the word midwife there comes the echo of the word abortionist. There are women in the field of midwifery who would hesitate at the induction of an abortion, but the great bulk of midwives receive a large portion of their income from abortion operations. This act in the eyes of the law is criminal, and in the light of morality unjustifiable, and is defined by a simple and almost unoffending word which when rightly interpreted, means destruction of life even though unborn, and destruction of life is defined by a much more objectionable word called murder.

The question has come up recently in the city of New York in relation to the licensing of midwives and the framing of a law giving them a legal status in the State. To the writer it would seem unquestionably wrong to license any set of people to do a particular class of work for which they are pre-eminently unfit either by personal ability or education, and it would seem that to license midwives as practitioners of midwifery, and give them the right to decide in cases of life and death when they have so rightly earned the reputation of abortionists, would be an act absolutely without justification to the originators of the law or to the legislators who made it. That it would be unquestionably of an advantage to the parturient woman who is to be cared for by the midwife that said midwife should be inspected and under the control of the Board of Health is a fact, but this control should be without giving to the midwife a legal standing.

At a conference recently held in the New York Academy of Medicine on the subject of midwives Miss F. Elisabeth Crowell presented the subject in a most exhaustive and complete manner. One of her statements is most surprising, that forty-two per cent. of the births in New York City were attended by midwives. This is probably accounted or by the large foreign population in this city. The apparent lack of untoward complications resulting in death either of the child or the mother in this large percentage is unquestionably due to the fact that in complicated or very critical cases the midwife calls in a physician, who gets the credit of the complication and has to sign either the stillbirth certificate or the death of the mother. Or, should the case be severe and beyond the reach of medical skill at the house of the patient, she is bundled off to a lying-in-hispital in a hurry and the hospital gets the credit of the complications. In this way I believe the rather favorable report so far as untoward complications is concerned in the paper above referred to is accounted for.

The midwife will unquestionably be employed until the campaign of education is carried on to such an extent that she is not called by those who appreciate that they are not receiving the proper care. It does not seem possible to eliminate the midwife, except by education of the people, neither does it seem right to institute legislation looking to the abolition of the midwife any more than it is right to license them. Let them be controlled by the Board of Health surveillance and subject to Board of Health law, as it is certainly class legislation to license any particular set of persons to practice any particular branch of medicine, granting them the power of life and death because they claim the ability to take care of the parturient woman. This would be pre-eminently as unjust as to grant to the Osteopath or the Christian Scientist the right to a title of any particular part of medicine. This has been the attitude of the medical societies from the time of the origin of organized medicine and any break would be a most serious and unwise step to take.—Ed. Annals of Gyn. and Ped., February, 1907.

